# Confronting inequality in the “new normal”: Hyper‐capitalism, proto‐socialism, and post‐pandemic recovery

**DOI:** 10.1002/sd.2196

**Published:** 2021-05-04

**Authors:** Tim Jackson, Peter A Victor

**Affiliations:** ^1^ Centre for the Understanding of Sustainable Prosperity University of Surrey Guildford UK; ^2^ Faculty of Environmental Studies York University Toronto Ontario Canada

**Keywords:** basic income, factor substitution, hyper‐capitalism, inequality, recovery, redistribution

## Abstract

Post‐pandemic recovery must address the systemic inequality that has been revealed by the coronavirus crisis. The roots of this inequality predate the pandemic and even the global financial crisis. They lie rather in the uneasy relationship between labor and capital under conditions of declining economic growth, such as those who have prevailed in advanced economies for almost half a century. This paper explores the dynamics of that relationship using a simple stock‐flow consistent (SFC) macroeconomic model of a closed economy. It examines in particular the role of two key factors—the savings rate and the substitutability (elasticity of substitution) between labor and capital—on the severity of systemic inequality under conditions of declining growth. The paper goes on to test the efficacy of three redistributive measures—a graduated income tax, a tax on capital and a universal basic income—under two distinct structural scenarios for an economy with a declining growth rate. We find that none of these measures is sufficient to control structural inequality when institutions aggressively favor capital over labor (hyper‐capitalism). Taken in combination, however, under conditions more favorable to wage labor (proto‐socialism), these same measures have the potential to eliminate inequality, almost entirely, even as the growth rate declines.

## 1 INTRODUCTION

The Covid‐19 crisis has laid bare the structural deficiencies that haunt late capitalism. Even before the pandemic struck, there was an increasing precarity at the heart of society. Its most devastating impact was on precisely those services that turned out to be critical for prosperity. Nurses, carers, cleaners, distribution and retail workers, and teachers: the frontline workers were both the first line of defense against the virus and those who bore the brunt of its impact. But these women and men were also those whose livelihoods and working conditions had become increasingly insecure in the preceding years.

It is tempting to trace this failing to the financial crisis of 2008. Were it not for those events, financial institutions would not have required massive bailouts—financed largely through public debt (Turner, 2015, Wolf, 2015). Without rising public debt, it would have been harder to justify the ideological turn to austerity, which derailed social investment and undermined the protections on labor (Basu & Stuckler, 2013). Without austerity, frontline workers would have been better prepared for what followed. Social welfare systems would have been stronger. Communities would have been more resilient.

There is a certain truth to this narrative. But an analysis which stops at that point has not properly done its job in uncovering the antecedents of a tragedy that has devastated the economy and overturned society in the course of the last year. The signs of precarity were already visible before the financial crisis (Jackson, 2019; Lazonick, 2017; Stiglitz, 2015). They had been there for decades, as the French economist Thomas Piketty pointed out in his bestselling book, first published in 2014. *Capital in the 21st Century* shone an unforgiving light on the rising inequality that had afflicted the most advanced economies in the world over the final decades of the 20th Century and the early years of the 21st.

Piketty's evidence was compelling. In the United States, for example, the richest 1% of the population received over 15% of the national income in 2015, a higher proportion than at any point since 1940 (Piketty, Saez, & Zucman, 2017). This trend had more than reversed the gains in equality witnessed in the immediate post‐war years. Between 1946 and 1980, the lowest income percentiles in the United States received the lion's share of the benefits from economic growth: average income growth in the lowest percentile was 6%, three times the average growth across the economy as a whole. Since 1980, it was increasingly the super‐rich who benefited from whatever growth the economy could provide. The average growth rate of the top 0.001% of the population was over 6%, allowing them to increase their post‐tax earnings by a factor of seven over the last three decades. The poorest 5% saw their post‐tax incomes fall in real terms over the same period (Piketty et al., 2017).

A key element in Piketty's analysis—and the principal concern of this paper—is a theoretical argument about the source of this inequality. Specifically, Piketty claimed that rising inequality is an inevitable consequence of a declining economic growth rate. This thesis is particularly challenging in the context of a secular stagnation such as the one recently discussed in advanced economies (Galbraith, 2014, Summers, 2014, Storm, 2017). It is also potentially problematic for those who are critical of growth for environmental or social reasons (D'Alisa, Damaria, & Kallis, 2014; Hickel, 2020; Kallis, Paulson, D'Alisa, & Demaria, 2020)—and we would count ourselves in that category (Jackson, 2021, 2017; Victor, 2019)—because it seems to suggest that doing without growth will inevitably lead to unpalatable social consequences: unless it is possible—perhaps through redistributive policy mechanisms—to offset these pernicious social dynamics. Some of those authors have also proposed policies to address these dynamics (Hartley, van den Bergh, & Kallis, 2020; Stiglitz, 2015).

Piketty's own suggestion for combatting systemic inequality is a tax on capital assets (Piketty, 2014). A heightening of conventional differential income tax rates might be another obvious policy candidate. A third potential policy, which has recently attracted a renewed interest, is the concept of a universal basic income (Gorz, 1999, RSA, 2015, Standing, 2017, Taylor, 2017). Sometimes also referred to as a citizen's income, a basic income is designed to provide people with a fundamental safety net under conditions of rising economic hardship. It has recently been posited, for instance, as a potential response to the threat of increased automation and declining job security (Frase, 2016; Pulkka, 2017; Varoufakis, 2016).

The aim of this paper is to explore the efficacy of such mechanisms in the face of a declining growth rate. In pursuit of that aim, we first set out Piketty's hypothesis in formal terms and describe briefly the structure and role of the model used in our analysis. In subsequent sections, we show how an unequal initial distribution of capital assets leads to widely different outcomes under different structural assumptions. We then explore the potential to mitigate rising inequality through three redistributive policy mechanisms (differential income tax, capital tax, and basic income), under two distinct structural scenarios for the evolution of the economy. One of these structural scenarios corresponds to a future of increased automation and digitalization, concentrated ownership, and vigorous protection of the interests of the owners of capital assets. The second corresponds to restraints on returns to capital and a more robust defense of the interests of wage labor. We conclude by discussing the implications of our findings.

## 2 TESTING THE “PIKETTY HYPOTHESIS”

Piketty hypothesized that rising inequality is an inevitable feature of a capitalist economy in the context of a declining growth rate. He advanced this hypothesis through the formulation of two “fundamental laws” of capitalism. The first of these (Piketty, 2014, p. 52 et seq) relates the capital stock (more precisely the capital to income ratio *β*) to the share of income *α* accruing to the owners of capital. Specifically, the first “fundamental law” of capitalism states that^1^:

(1) α=rβ,

where *r* is the rate of return on capital. Since *β* is defined as *K/Y* where *K* is capital and *Y* is the net national income, it is easy to see that this “law” is in fact an *accounting identity*:

(2) αY=rK.

Formally speaking, the income accruing to capital equals the total capital multiplied by the rate of return on that capital. Though this “law” on its own does not force the economy in one direction or another, it provides the accounting framework within which the evolution of relationships between capital, income, and rates of return takes place. For instance, it can be seen from this identity that for any given rate of return *r* the share of income accruing to the owners of capital rises as the capital to income ratio rises.^2^

The second “fundamental law of capitalism” (op cit: 168 et seq; see also Piketty, 2010) states that in the long run, the capital to income ratio *β* tends towards the ratio of the savings rate s to the growth rate *g*, that is:

(3) $$\beta \to \frac{s}{g}\;\mathrm{as}\;t\to \infty .$$

It is in putting Equations (2) and (3) together that we encounter the challenge inherent in Piketty's argument. Specifically, capital's share of income *α* would be governed by the following relationship:

(4) $$\alpha \to r\frac{s}{g}\;\mathrm{as}\;t\to \infty .$$

In other words, as growth declines, the rising capital to income ratio *β* leads to an increasing share of income *α* going to capital and a declining share of income going to labor. Unless the distribution of capital is itself entirely equal this relationship therefore presents the spectre of a rapidly escalating level of income inequality. Differential savings rates—in which higher income earners save proportionately more than lower income earners (or, equally, where there are lower propensities to consume from capital than from income)—would reinforce these inequalities further by allowing the owners of capital to accumulate even more capital and command even higher wages. The superior power of capital (op cit 22–25) then precipitates a rising structural inequality. As Krusell and Smith (2014, p. 2) point out, Equation (4) is “alarming because it suggests that, were the economy's growth rate to decline towards zero, as Piketty argues it will, capital's share of income could increase explosively.”

In fact, as we have shown in a previous paper (Jackson & Victor, 2016), this alarm is justified only under certain conditions associated with the structure of the economy. In particular, capital's share of income is highly responsive to the elasticity of substitution *σ* between the “factors of production” capital and labor. In the earlier paper, we developed a simple, five‐sector^3^ model of Savings, Inequality and Growth in a Macroeconomic Account (SIGMA) to explore the behavior of both capital's share of income and the implications of this on inequality as the growth rate declines to zero.^4^

A key feature of the SIGMA model is a division of the population into two household subsectors, which for illustrative purposes we nominate as “workers” and “capitalists.” Initially, our simulations assume complete parity between these two sectors, in relation to population, wage income, savings behaviors, and the ownership of capital assets. In later simulations, we relax these assumptions to reflect the unequal ownership of capital in society and also to explore the potential for differences in the savings behaviors of the respective household sectors. The model as a whole was loosely calibrated on the basis of an advanced economy “similar to” the UK or Canada, say. That is, the broad magnitudes of macro‐economic aggregates in SIGMA are chosen to reflect values typical for these countries; the initial split between wages and profits is similar; the expenditure basis of the SIGMA economy is comparable and the initial savings rates are based on empirical data in the case study countries (Jackson & Victor, 2016, Appendix 1, p. 218).

The SIGMA model allows us to assess the implications of a slowdown of growth on (a) capital's share of income and (b) the distribution of incomes in the economy. By adding a government sector to the model, we are also able to explore the potential to mitigate regressive impacts through fiscal redistribution mechanisms. The inclusion of a banking sector allows us to establish clear relationships between the real and the financial economy. Most importantly for our purposes, we can explore the impact of a decline in the growth rate over time on the income shares from capital and labor through an endogenous rate of return, *r*, on capital.

To achieve this we assume, as Piketty also did (Piketty, 2014, pp. 213–214), that the return to capital is given by the marginal productivity of capital, which we denote by *r*_*K*_. This assumption only works under conditions where there are no structural features, which might lead either capital or labor to extort more than their “fair” share of the output from production. In a sense, this assumption is a conservative one for us, to the extent that conclusions about inequality are stronger in imperfect market dynamics. Under conditions of duress, in which the owners of capital receive a rate of return *r* greater than the marginal productivity of capital *r*_*K*_, our conclusions about any inequality which results from declining growth rates will be reinforced. Conversely, of course, we must beware of making too strong assumptions about the potential to mitigate inequality, in any situation, in which the owners of capital have greater bargaining power than wage labor.

Under this assumption, the rate of return on capital can be calculated from a constant elasticity of substitution (CES) production function^5^ of the form first developed by Arrow, Chenery, Minhas, and Solow (1961) in which output, *Y*, is given (cf Jackson & Victor, 2016, p. 210, eq. [20]) by:

(5) $$Y\left(K,L,\sigma \right)={\left(aK^{\frac{\left(\sigma -1\right)}{\sigma }}+\left(1-a\right){\left(\mathrm{AL}\right)}^{\frac{\left(\sigma -1\right)}{\sigma }}\right)}^{\frac{\sigma }{\left(\sigma -1\right)}},$$

where *σ* is the elasticity of substitution between labour and capital, *a* (as described by Arrow et al. (1961) is a “distribution parameter” and *A* is the coefficient of technology‐augmented labour, which we assume changes over time according to the change in labour productivity in the economy.^6^ With a little effort, it can be shown via partial differentiation of Equation (5) with respect to *K* that the marginal productivity of capital *r*_*K*_ is given by:

(6) $$r_{K}=\frac{\partial Y}{\partial K}=a\beta^{\frac{-1}{\sigma }},$$

where *β* is the capital to income ratio.^7^ This relationship can now be used to derive the return to capital, *r*_*K*_ *K*, through:

(7) $$r_{K}K=a\beta^{\frac{-1}{\sigma }}K.$$

Taking *Y* to be the national income (net of depreciation), and using Piketty's first law of capitalism (Equation [2] above) it can be shown that capital's share of income *α* is given by:

(8) $$\alpha =a\beta^{\frac{\sigma -1}{\sigma }}.$$

Equation (8) allows us to explore explicitly what happens to capital's share of income under different assumptions about the elasticity of substitution *σ*. For *σ* > 1, (and assuming that the capital to income ratio is greater than one) capital's share of income is an increasing function of the capital to income ratio. As the capital to income ratio rises, capital's share of income increases. Conversely, when *σ* < 1, capital's share of income is a decreasing function of the capital to income ratio. As the share of capital to income rises, capital's share of income decreases.

**FIGURE 1:**
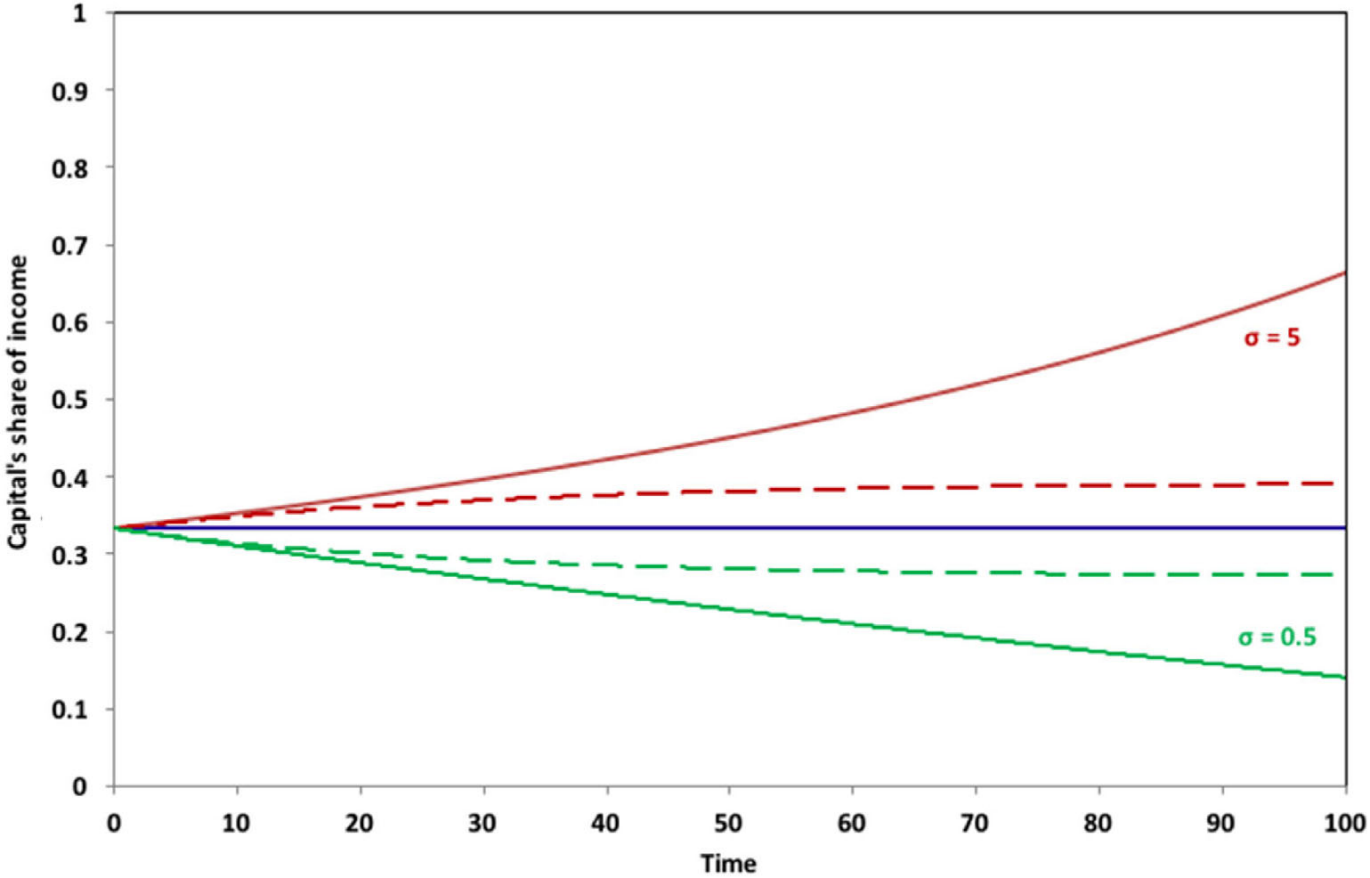
Long‐term behavior of capital's share of income as σ varies and g → 0. Solid lines indicate scenarios where the savings rate remains unchanged over the course of the run; dashed lines indicate scenarios where the savings rate falls to zero as the growth rate declines. For comparison with similar results in Jackson & Victor (2016), Figure 4, note that the results in the earlier paper were restricted to the case where the savings rate remained constant [Colour figure can be viewed at wileyonlinelibrary.com]

Figure 1 illustrates the outcome of this analysis, for three different values of *σ*: 0.5, 1, and 5. When the elasticity of substitution *σ* has a value of 5, capital's share of the total income increases, in accordance with Equation (8). Specifically, under a scenario where the savings rate remains constant as the growth rate declines (shown by the solid upper line in Figure 1), capital's share of income doubles over the length of the run. Conversely, however, with an elasticity of substitution less than 1, capital's share of income declines over the period, in spite of the fact that both *s*/*g* and *rs*/*g* go to infinity. With *σ* equal to 0.5, and with the savings rate held constant (the solid lower line in Figure 1), capital's share of income has more than halved over the course of the run.

Mathematically, we see from Equation (8) that when *β* is increasing and its exponent is greater than 0, then the share of income going to capital will continue to increase over the run. Likewise, when *β* is increasing and its exponent is less then 0, which is the case for *σ* = 0.5, then the share of income going to capital must continually decrease over time as shown in the solid lower line in Figure 1. At *σ* = 1, which is the Cobb Douglas case, the decline in the rate of return to capital always exactly offsets the rise in the capital to income ratio, and capital's share of income remains constant.

**FIGURE 2:**
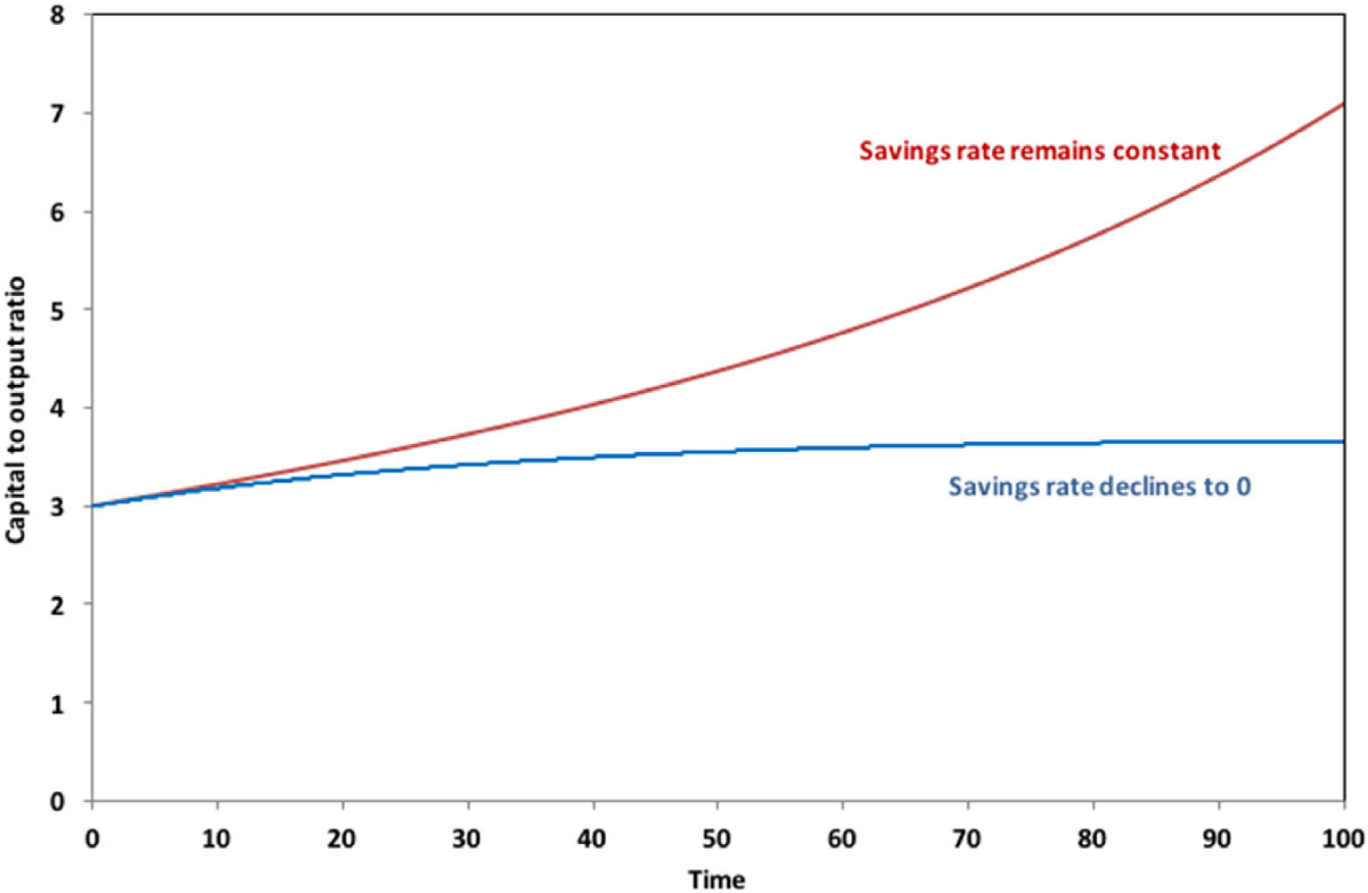
Long‐term behavior of the capital‐to‐output ratio as g → 0. under different savings rate assumptions [Colour figure can be viewed at wileyonlinelibrary.com]

A result similar to the solid lines in Figure 1 was already shown (as Figure 4) in Jackson & Victor, 2016. In the current paper, we extend the analysis from the earlier paper to the case where the savings rate is not held constant. Specifically, we see that the broken lines in Figure 1 and the lower line in Figure 2 represent a rather different case, in which the savings rate declines to zero alongside the growth rate. In this case, as Figure 2 shows, the capital to output ratio *β* converges to a constant value. By the end of the run, there is no net investment and no more growth in the economy. Both the output and the capital stock are then unchanging.

Interestingly, under these circumstances, capital's share of income *α* remains firmly bounded. As the dotted lines in Figure 1 illustrate, when *σ* = 5, *α* converges to a value that is slightly higher than the initial share of capital, and when *σ* = 0.5, to a value that is slightly lower than the initial share. Mathematically, we can see from Equation (8) that if *β* converges to a constant value *β*_100_, as *g* goes to zero, then so does the capital share *α*, with values given by $a\beta_{100}^{0.8}\;\text{and}\;a/\beta_{100}$, when *σ* = 5 and 0.5, respectively. For the values assumed in the SIGMA model, capital's share of income after 100 periods moves just ±6 percentage points from the initial capital share of 33%. More importantly, once the savings rate and the growth rate have both fallen to zero, these shares remain constant. There is no indication of an “explosive” increase in the share of income going to capital, even under high elasticities of substitution between labor and capital.

It is now possible to see that Piketty's hypothesis of an inevitable dramatic increase in inequality arising from a decline in the growth rate holds only under particular circumstances. Clearly, there are some instances, such as the case shown by the upper solid line in Figure 1, where an increasing proportion of the national income goes to capital and a declining (indeed eventually disappearing) proportion goes to labor. Here, the fear of an explosive increase in inequality is valid. Elsewhere and, in particular for cases where the savings rate declines to zero alongside the growth rate, the share of income going to capital is either firmly bounded or else declines continually, avoiding the dangers presented by Piketty's hypothesis.

**FIGURE 3:**
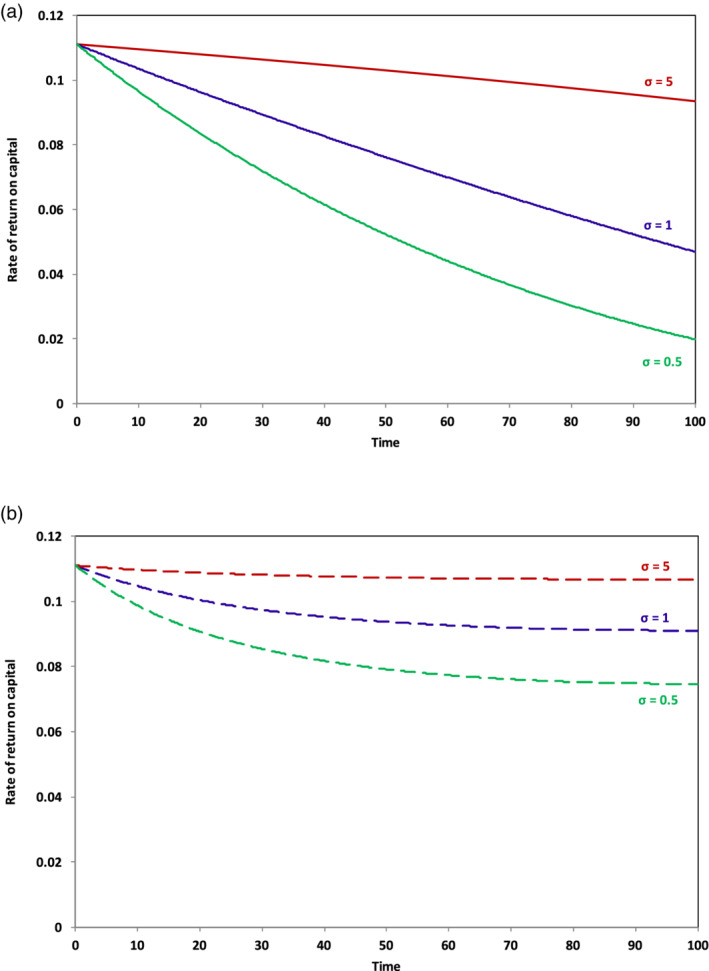
Long‐term behavior of the rate of return on capital as g → 0. Solid lines (a) indicate scenarios where the savings rate remains unchanged over the course of the run; dashed lines (b) indicate scenarios where the savings rate falls to 0 as the growth rate declines [Colour figure can be viewed at wileyonlinelibrary.com]

The behavior of the savings rate as the growth rate declines is going to depend in practice on the confidence of investors in being able to protect the return on capital. This will depend in its turn on the relative power of capital and labor in the economy. Our model allows us to gain some insight into this dynamic be exploring an endogenous rate of return on capital. Figure 3a shows the rates of return on capital (for different values of *σ*) when the savings rate is held constant in the model and the growth rate declines. Figure 3b shows the rates of return when the savings rate goes to zero over the run.

In the former case, we can see that the rate of return on capital falls more or less precipitously, depending on whether *σ* is (respectively) lower or higher. For *σ* = 0.5, the rate of return on capital falls from around 11% at the beginning of the run to around 2% at the end of the run. With low substitutability between labor and capital, it is not possible for the owners of capital to increase revenues by lowering costs and the effect of investment is simply to push up the capital to output ratio in the economy (Figure 2) without a corresponding growth in demand. By the end of the run, when the growth rate has (by construction) fallen to zero, net investment is simply soaking income away from consumption and government expenditure, building capital for no apparent reason. The situation here is essentially the one characterized by Keynes in the last chapter of the *General Theory* as “the euthanasia of the rentier,” in which a persistent oversupply of savings looking unsuccessfully for profitable investment leads to a progressive decline in the rate of return on capital (Keynes, 1936). When there is high substitutability between capital and labor (*σ* = 5, the upper solid line in Figure 3a), there is more chance for private investors to stabilize their profits, leading to an increasingly high share of income going to capital (as shown by the upper solid line in Figure 1). This is the one case, which fits the Piketty analysis most clearly and gives rise to the biggest fears about runaway inequality.

Figure 3b shows a rather different pattern to the long‐term behavior of the rate of return, under conditions in which, rather than remaining constant, the savings rate falls to zero alongside the declining growth rate. Under these circumstances, the return to capital is more resilient. Some fall is still clearly visible in Figure 3b, but this stabilizes relatively quickly and rates of return across the range of values of *σ* are considerably higher than in the case where the savings rate remains constant. Note that when the growth rate has declined to zero, a zero rate of net savings is consistent with a constant capital‐to‐output ratio. In fact, this assumption (of constant capital‐to‐output) is widely held in post‐Keynesian models (see e.g., Godley & Lavoie, 2007, Appendix 2) and to some extent justified on the basis of empirical data (e.g., ONS, 2017). We will return to the question of the likely behavior of the savings rate under different conditions in the Discussion section. The key point to note here is that, when the savings rate declines rather than remaining constant, capital's share of income is clearly bounded—in stark contrast to the Piketty hypothesis.

## 3 SAVINGS AND THE DISTRIBUTION OF INCOMES

The functional distribution of income between labor and capital tells us little about the actual distribution of incomes in the population without some account of the ownership of capital assets. Under the conditions of our reference case, both income and wealth are equally distributed between workers and capitalists. There is no inequality in such a society, whatever happens to the share of income going to capital. Clearly, this is not very realistic as a depiction of capitalist society. One of the things we know for sure, not least from Piketty's work, is that the distribution of both wealth and incomes is already skewed in modern societies, sometimes excessively. In fact, as we next demonstrate, inequality in incomes can arise simply from differential savings rates between different household sectors.

**FIGURE 4:**
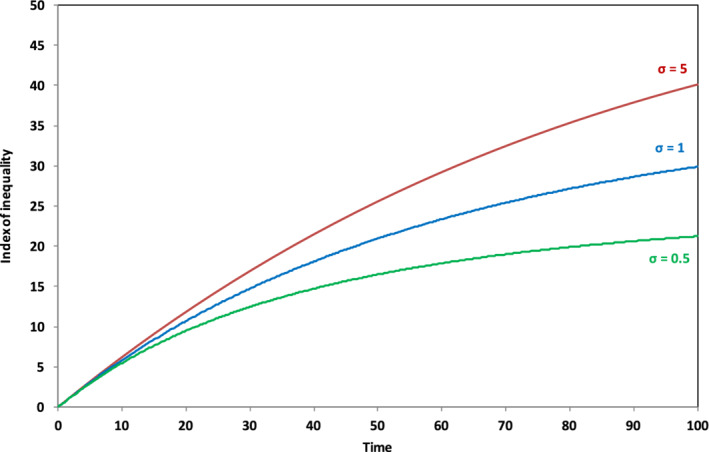
Income inequality arising from differential savings rates (*g* = 2%) [Colour figure can be viewed at wileyonlinelibrary.com]

Let us suppose that—for whatever reason—the savings rate of “workers” is lower than the savings rate across the economy as a whole—say 5% as opposed to 8%, with the savings rate of “capitalists” rising to compensate. Figure 4 shows that this apparently trivial model innovation immediately introduces income inequality. The index of inequality shown in Figure 4 is constructed by taking the ratio of the disposable income of capitalists to the disposable income of workers subtracting one and multiplying by 100. The vertical axis in Figure 4 thus represents the percentage increase of capitalist incomes above worker incomes. By the end of the run and without any decline in the growth rate, the disposable incomes of capitalists are more than 40% higher than the disposable income of workers. This is a fascinating insight into the structural dynamics through which capitalism has an in‐built tendency toward a divergence of incomes (Kalecki, 1939, Kaldor, 1955, Galbraith, 2013).

**FIGURE 5:**
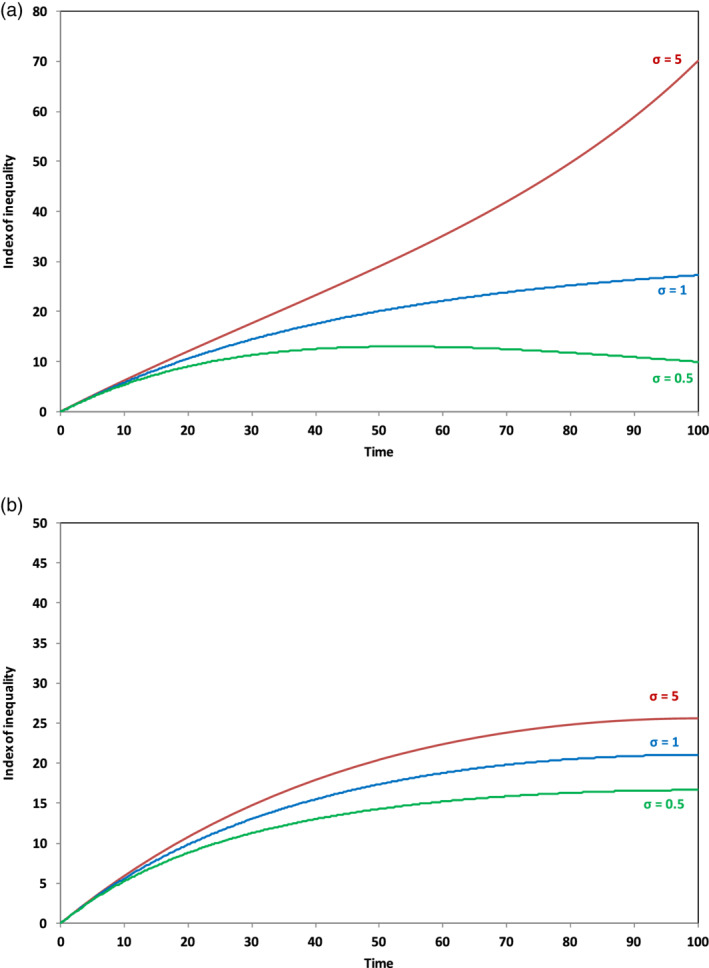
(a) Income inequality arising from differential savings rates (*g* → 0, *s* = 8%). (b) Income inequality arising from differential savings rates (*g* → 0, *s* → 0) [Colour figure can be viewed at wileyonlinelibrary.com]

Under conditions of slowing growth (Figure 5), as we might expect from the previous analysis, the evolution of inequality is dependent on two key factors: the elasticity of substitution *σ* and the behaviour of the savings rate *s*. Suppose first that the savings rate remains constant. Then, for high *σ*, that is, for high substitutability of labor for capital (Figure 5a), the inequality between capitalists and workers is exacerbated. When *σ* = 5, capitalist incomes are over 70% higher than worker incomes by the end of the scenario. By contrast, the situation is improved for low *σ*. Capitalist incomes are less than 10% above worker incomes at the end of the run when *σ* is equal to 0.5 and inequality is declining, largely because of the steep decline in the rate of return on capital (Figure 3a). For the case where the savings rate declines alongside the growth rate (Figure 5b), the results are much less differentiated. For each value of *σ*, inequality remains bounded and, perhaps surprisingly, inequality is lower for each value of *σ* than the case with a 2% growth rate (Figure 4). In these circumstances, in other words, far from increasing inequality, growth rate stagnation may, under certain conditions, be accompanied by reduced inequality.^8^

**FIGURE 6:**
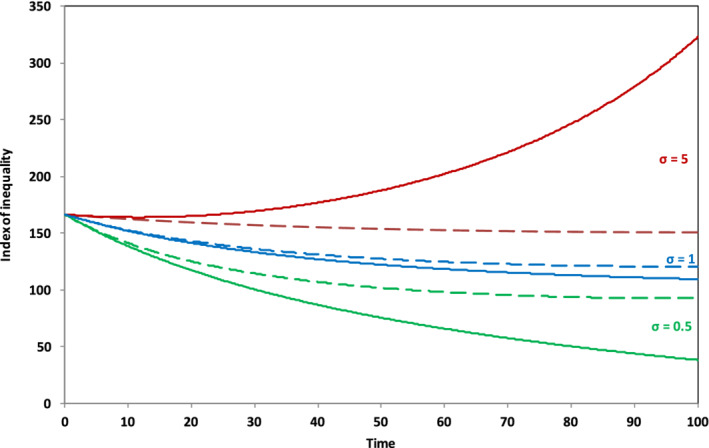
Income inequality with skewed initial ownership and differential savings. Solid lines indicate scenarios where the savings rate remains unchanged over the course of the run; dashed lines indicate scenarios where the savings rate falls to zero as the growth rate declines. As with Figure 1, Figure 6 also extends the results obtained in the previous paper (Jackson & Victor, 2016) by including the case where the savings rate declines to zero alongside the case where the savings rate remains constant [Colour figure can be viewed at wileyonlinelibrary.com]

The inequality shown in Figures 4 and 5 arises simply from changing the savings rates, assuming a completely equal distribution of income and capital at the outset. Figure 6 illustrates what happens, when the initial distribution of assets is unequal. For the purposes of this illustration, we assume that capitalists comprise only 20% of the population but own 80% of the wealth—a proportion not massively unrealistic from the perspective of today's global distribution (ONS, 2014; Oxfam, 2015). We also assume (rather conservatively) that the distribution of wages remains proportional between the two groups, despite the skewed distribution in asset ownership: capitalists earn 20% of the wages and workers earn 80%. Finally, we maintain the savings rate differential between workers and capitalists assumed in the previous experiment.

The first thing to note from Figure 6 is that capitalist incomes are immediately around 166% higher than worker incomes at the start of the run because of the profits achievable from greater ownership of capital assets. What happens subsequently depends crucially on the evolution of the savings rate *s* and the value of σ.

The dependency is complex. Specifically, when the overall rate of savings across the economy is conserved through the run (shown by the solid lines in Figure 6), the level of income inequality is highly sensitive to the elasticity of substitution between labor and capital. With high σ (the upper solid line in Figure 6), capitalists can protect their return on capital by continually substituting capital for labor and suppressing wages. This leads to a steeply rising income inequality—somewhat similar to the scenario envisaged by Piketty. For low σ under conditions of constant saving, however, (the lower solid line in Figure 6), capitalists are unable to substitute away from labor and as the growth rate slows down, rates of return to capital fall and capitalist income is moderated, leading to a significant decline in income inequality. When the savings rate declines alongside the growth rate (the dotted lines in Figure 6), then outcomes are considerably less sensitive to the value of σ. It is notable immediately that, in this case, income inequality is bounded and falling over the course of the run even for high σ.

## 4 POLICY EXPERIMENTS

We are now in a position to explore the potential of fiscal policy measures to reduce inequality. We test these measures in two distinct scenarios, reflecting different assumptions about economic structure, based on the earlier discussion. In both scenarios, we assume the same initial distribution of capital as explored in the previous section, namely 80% of the wealth is owned by capitalists and 20% by workers. There is wage parity across the two income groups, but, as before, workers save at a slower rate than capitalists. Following the discussion above, we define structural differences in the two scenarios according to the values of (a) the elasticity of substitution between labor and capital and (b) the savings rate across the economy. In both scenarios, we assume that the growth rate declines to zero.

- Scenario 1 is a form of *hyper‐capitalism* in which there is a concerted effort to maintain a high savings and investment rate and a high elasticity of substitution between labor and capital. In this scenario, as we have noted above, the rate of return falls only slightly (the uppermost line in Figure 3a), providing an incentive for continued saving, and the economy is becoming increasingly capital intensive. Inequality (absent of policy) in this scenario is described by the upper solid line in Figure 6.
- Scenario 2 is a form of *proto‐socialism* ^9^ in which labor is protected against the aggressive interests of capital as the growth rate declines. There is a low substitutability between labor and capital and the savings rate declines to zero alongside the growth rate. The rate of return on capital falls initially, to a level that is lower than in Scenario 1, but subsequently stabilizes as the capital to output ratio converges to a constant value. Income inequality (absent of policy) is described by the lowest broken line in Figure 6.

We then consider three specific fiscal interventions:

- A graduated income tax regime in which incomes above the average worker income are taxes at twice the level of taxation on worker incomes: specifically, income in excess of worker income is taxed at 50% (as opposed to a 25% tax on income at or below the average worker income);
- A small tax on household wealth: specifically, household assets are taxed at the rate of 2.5% of the value of net assets;
- A citizen's income provided to every citizen across the economy equally: specifically, a universal basic income equivalent to 10% of the average worker salary is provided to everyone, whether employed or unemployed, worker or capitalist.

All three measures are introduced gradually in the model over the first 20 periods. After that point, the tax rates are held constant. The first measure is relatively conventional. Graduated tax regimes are common practice in most advanced economies and many less advanced economies. A higher tax rate on income over a certain threshold is commonly used as a way of redistributing income and providing social security for the poorest in society. The second proposal of a tax on capital assets is the one suggested by Piketty (2014) to offset the rise in inequality that he assumes will take place when there is a declining growth rate. Though less common in practice it has a relatively long pedigree in economic thought, for instance in Henry George's proposals for a land tax.^10^

The final suggestion is the universal basic income—sometimes called a citizen's income or a social dividend. This idea too has a long pedigree. Thomas More included the idea in his 1,516 description of *Utopia* (More, 1963). Over the years, it has been advocated by a wide range of economists and political theorists (Gorz, 1999; McKay, 2001; Meade, 1988; Wright, 2005) and has been revived recently by a variety of commentators from across the political spectrum (JRF, 2015; Murray, 2008; RSA, 2015; Varoufakis, 2016). Numerous pilot schemes have been implemented—in Finland (Guardian, 2017; 2018), in Alaska (BIEN, 2015) and elsewhere (Colombino, 2019).^11^

To model the citizen's income in SIGMA, we adopted a proposal similar to those suggested by Wright (2005) and Varoufakis (2016) in which universal basic income is funded through a social dividend paid from the ownership of “common stock”—that is to say equities purchased and held on behalf of the public by the nation state.

This is of course a rather striking departure from a pure capitalist model in which the ownership of productive assets is assumed to be held in private hands. But it responds explicitly to the underlying inequality in the ownership of assets. It also draws justification from the idea that profit is a form of social contract (Varoufakis, 2016), which should reflect, at least in part, the investment made by the state in education, in primary research and in the development the means of production itself. As Mazzucato (2018) has pointed out, every key innovation in Apple's iPhone was funded by the US government. The assumption that only Apple's shareholders should benefit from profits on sales of the iPhone is therefore a distortion of the social contract in favor of the owners of capital and against the interests of the public.

**FIGURE 7:**
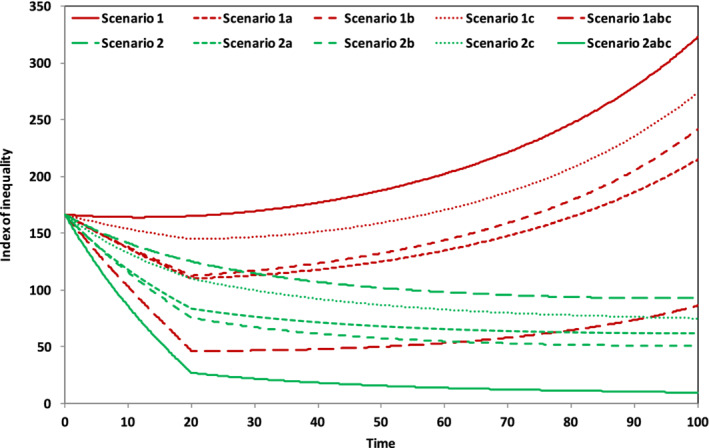
Tackling structural inequality through fiscal policy [Colour figure can be viewed at wileyonlinelibrary.com]

In our model, the state purchases equities on a year on year basis, following an adjustment model in which equities are purchased if the gap between the citizen's income paid and the dividend from common stock is greater than zero and sold if the gap is less than zero. This structure allows the government both to stabilize the level of the citizen's income and also to balance its equity holdings over the longer term.^12^ Figure 7 shows the results of the simulations.

There are two groups of results each associated with one of the scenarios described above. Broadly speaking, the upper set of lines (shown in red) in Figure 7 refers to Scenario 1 and the lower set of lines (shown in green) refers to Scenario 2, although the lowest line in Scenario 1 overlaps with several of the lines in Scenario 2. The broad rule of thumb that distinguishes these two scenarios visually is that, following period 20 when the level of intervention is no longer increasing, inequality increases for all variants of Scenario 1 and declines for all variants of Scenario 2.

More specifically, the uppermost line of all shows the income inequality in the reference case for Scenario 1 (i.e., before fiscal intervention). The broken red lines below this solid upper line illustrate the impact of each intervention in turn, in terms of reducing income inequality. The lowest broken line from this Scenario, which first dips sharply down as the measures are introduced and then increases throughout the rest of the run, shows the effect of implementing all three measures together. It illustrates that these policy measures on their own, even taken together, are insufficient to contain inequality in the longer term. Once the level of intervention has stabilized, income inequality returns to an increasing path.

By contrast, the lower set of (green) lines associated with Scenario 2 show declining income inequality throughout the run. The uppermost (broken) line from this set describes the reference case and is identical to the lowest broken line in Figure 6. The next three green lines illustrate the impact of each of the three policy interventions, taken on their own. The lowest solid (green) line in Figure 7 shows the implications of implementing all three measures together in Scenario 2. At the end of the run, the per capita income of capitalists is less than 10% higher than the per capita income of workers. In other words, inequality has almost entirely been eliminated.

As regards the relative impact of the individual measures, it is to be noticed that the basic income has the least impact on inequality (at this level of implementation). This is not particularly surprising, since the basic income is given equally to both worker and capitalist households. The main distributive effect takes place therefore by removing productive assets from private ownership and reducing the returns to capital available to private asset owners. The relative effectiveness of the graduated income tax and the capital tax switch switches between the two scenarios. In Scenario 1, an income tax is more effective (at the chosen level). In Scenario 2, the capital tax becomes the more effective instrument.

Of course, it is difficult to make hard and fast conclusions about relative effectiveness when the levels at which the various measures are applied have been chosen fairly arbitrarily. But some assessments are possible on the basis of political acceptability. For instance, an income tax band higher than 50% might struggle for acceptability in some advanced economies (such as the UK). Imposing an even higher level of income taxation might therefore prove difficult. Although the level of capital tax is rather low (2.5%), the actual transfer of funds from private individuals is relatively large and may again suffer from political resistance. A citizen's income of 10% of the unadjusted worker income is lower than has been proposed by some advocates. Citizen's Income Trust proposals are around the 20% mark for example. But the Alaskan dividend is closer to this level. Moreover, higher levels of social dividend run into another kind of problem of political acceptability. At the end of the run, with a citizen's income of 10%, the state already ends up owning between 20% and 30% of the nation's productive assets. Doubling that income more or less doubles that level of public ownership. Clearly at that point, the economy begins to look very unlike any capitalist economy of the last half a century or so.

## 5 DISCUSSION

The rising inequality observed within advanced economies over recent decades may, as Piketty has suggested, be a structural feature of capitalism in the 21st Century. It is not, however, an inevitable feature of an economy with a declining growth rate. Rather, as we have shown in this paper, the progress of inequality depends crucially on the institutional context within which a decline in the growth rate takes place.

Under certain conditions, it is entirely possible for income inequality to rise precipitously as the growth rate declines. However, we have also established that there is *no inevitability* that a declining growth rate leads to explosive (or even increasing) levels of inequality. Even under a highly skewed initial distribution of ownership of assets, it is entirely possible to envisage scenarios in which income inequality declines over the longer term, even without intervention from progressive taxation policies.

The two key structural factors, which determine the evolution of inequality under a declining growth rate, are (1) the savings rate and (2) the elasticity of substitution between labor and capital. Depending on the configuration of these factors, two radically different futures may emerge. Under one future, which we have described here as “hyper‐capitalism” (Scenario 1), a constant savings rate and high substitutability between capital and labor lead to accelerating inequality, even under a progressive combination of redistributive measures. Under another kind of future, which we describe as proto‐socialism (Scenario 2), a declining savings rate and low substitutability between capital and labor, lead to declining inequality, which in combination with progressive redistributive policies, have the potential to eliminate inequality almost completely.

Turning next to redistributive policies, the most striking finding from our model is that even relatively progressive policies, which impose a combination of higher differential tax rates, taxes on capital and a basic income (funded from returns to capital) remain ineffective in the long run in bringing down structural inequality under the hyper‐capitalism described in Scenario 1. Higher capital to income rates, constant savings rates, and the rigorous protection of rates of return on investment lead inevitably to the “explosive” inequality highlighted by Piketty, and the best efforts of progressive fiscal policies are unlikely to be able to halt this rise. Even the much vaunted “solution” of a basic income fails to curb the inevitable rise in inequality under such conditions (Scenario 1). In the “proto‐socialism” of Scenario 2, on the other hand, this same combination of measures is strikingly effective. By the end of the run (Figure 7), inequality between capitalists and workers is almost entirely eliminated.

Hyper‐capitalism is likely to emerge in a world where labor is increasingly (and easily) substituted with capital and the interests of the owners of capital are privileged over the rights of workers. These privileges encourage capitalists to continue to save even as the growth rate declines, leading to a rising capital to output ratio and an escalating inequality. Such a scenario could, for example, accompany a world in which an aggressive drive towards automation or the implementation of artificial intelligence (AI) by monopolistic companies removes the need for wage labor across large swathes of the economy. Failure to protect the livelihoods of the immiserated work force facilitates continued savings and investment by asset owners. By the same token, it concentrates incomes (and wealth) increasingly in a minority of the population, leading to the kinds of dystopian trends in inequality illustrated in Scenario 1.^13^

Proto‐socialism on the other hand aims for strong institutions to protect the rights of workers, introduce a job guarantee, and establish an adequate minimum wage. Such interventions slow down the substitution of capital for labor. Attempts by capitalists to maintain a constant savings rate under these conditions lead (Figure 3a) to a dramatic collapse in the rate of return on investment, and a partial reversal in the relative fortunes of workers and capitalists. Faced with the prospect of declining rates of return, these conditions are more likely to lead to a decline in the rate of savings (Scenario 2) and a reduction in the capital intensity of the economy, features that will reinforce a more equal distribution of incomes.

In short, proto‐socialism is likely to involve a transition away from resource‐intensive mass production processes and toward the evolution of an economy of quality and service (Jackson, 2017). It might well also involve institutional innovations which better represent the interests of workers in the management of firms (Ferrera, 2017), better distribute the rewards of innovation to the populace (Varoufakis, 2016) and allow government to operate as an “employer of last resort” (Minsky, 1986).

It will not have passed unnoticed that the sectors that emerge stronger under proto‐socialism are precisely the labor‐intensive sectors associated with care, distribution and maintenance—the frontline services of the pandemic—described at the beginning of this paper. Other labor‐intensive sectors such as those associated with crafts, creativity, and community‐based recreation and leisure (Jackson, 2021) are also likely to flourish under these conditions. Proto‐socialism, in other words, could provide a robust basis for a post‐pandemic recovery—even under conditions of low‐growth.

In summary, the idea that rising income inequality is an inevitable consequence of declining growth rates is clearly wrong. On the contrary, the “new normal” might equally be headed towards lower income inequality and greater stability with respect to the substitution between labor and capital. The choice lies in the underlying structure of economic relations and, in particular, of the relations between labor and capital.

## Endnotes

^1^ In what follows, we suppress specific reference to time‐dependency of variables except where absolutely necessary. Thus all variables should be read as time dependent unless specifically denominated with a subscripted suffix 0. Occasionally, we will have reason to use the subscripted suffix (−1) to denote the first lag of a time‐dependent variable.

^2^ We will see later that the ceteris paribus clause relating to constant *r* here is important. In fact, the rate of return will typically change as the capital to income ratio rises; and to the extent that this ratio declines with increasing *β*, it can potentially mitigate the accumulation of the capital share of income.

^3^ The model comprises two households sectors (“workers” and “capitalists”), a nonfinancial firms sector, a banks sector and a government sector.

^4^ A user‐version of the SIGMA model is available online at https://www.cusp.ac.uk/projects/systems/sigma-model/ to allow the interested reader to explore the scenarios generated here and to conduct their own scenarios.

^5^ In fact, the inclusion of a broadly neoclassical production function is an unusual way to model the production relationships in an SFC model. Coming broadly from a Keynesian perspective such models tend to eschew aggregate production functions for all the well‐known reasons (Cohen & Harcourt, 2003; Robinson, 1953). However, retaining this aspect of Piketty's analysis allows us to compare our findings more directly with his.

^6^ It can be shown that, for the special case *σ* = 1, this CES function reduces to the familiar Cobb–Douglas production function *Y* = *K* ^*a*^(*AL*)^1 − *a*^. The introduction of an explicit elasticity variable allows for a more flexible exploration of the production relationship under a variety of different assumptions about the elasticity of substitution between labour and capital.

^7^ Note that as *σ → 1*, this relationship returns to the “first law” of capitalism (Equation [1]) with *a = α*. In other words, under an assumption of unit elasticity of substitution between capital and labour as in the Cobb Douglas function, the constant *a* is given by the share of income to capital *α*.

^8^ It is however worth highlighting here, as pointed out by one of our reviewers, that a “degrowth” world, in which growth rates were consistently negative over some extended period of time would create even more punishing conditions, possibly even requiring a negative elasticity of substitution between capital and labour. It is beyond the scope of this paper to explore such an economy in detail. But Hartley et al. (2020) do include redistributive policies relevant for this more general case.

^9^ The terms proto‐socialism was first used by the philosopher Rudolph Bahro (1977) to describe the emergence of socialist responses to late capitalism in Eastern Europe in the middle of the 20th Century. We use it here to refer to a partial amelioration of late capitalism to offset its structural inequalities.

^10^ Interestingly, this type of measure was proposed (at least in pilot form) by at least two political parties in the most recent UK general election in June 2017.

^11^ See https://www.visualcapitalist.com/map-basic-income-experiments-world/ for a summary of initiatives from 1960 onwards.

^12^ SIGMA has the potential for equity purchases to be made either from tax receipts or from deficit spending. The results shown in this paper are for purchases made through deficit spending. Since common stock is an asset of the government, it is to be noted that this kind of purchase does not in itself change the net debt to equity ratio of the nation.

^13^ An interesting alternative example might be the additional capital investment needed for the transition to a low‐carbon economy. More generally, the investment needed to sustain and enhance ecological assets or biological diversity might lead to a rising capital‐to‐output ratio.

## References

[sd2196-bib-0001] Arrow, K. , Chenery, H. , Minhas, B. , & Solow, R. (1961). Capital‐labor substitution and economic efficiency. The Review of Economics and Statistics, 43(3), 225–250.

[sd2196-bib-0002] Bahro, R. (1977). In D. Fernbach (Ed.), The alternative in Eastern Europe. London, England: NLB.

[sd2196-bib-0003] Basu, S. , & Stuckler, D. (2013). The body economic – Why austerity kills. London, England; New York, NY: Basic Books.

[sd2196-bib-0004] BIEN . (2015). Alaska, USA: 2015 dividend estimated to be near highest ever. Basic Income Earth Network. Retrieved from http://basicincome.org/news/2015/08/alaska-usa-dividend-amount-estimated-to-be-near-highest-ever/.

[sd2196-bib-0007] Cohen, A. , & Harcourt, G. (2003). Retrospectives: Whatever happened to the Cambridge capital theory controversies? Journal of Economic Perspectives, 17(1), 199–214.

[sd2196-bib-0008] Colombino, U. (2019). Is unconditional basic income a viable alternative to other social welfare measures? IZA World of Labor, 2(128), 1–11.

[sd2196-bib-0009] D'Alisa, G. , Damaria, F. , & Kallis, G. (Eds.). (2014). Degrowth: A vocabulary for a new era. London, England: Routledge.

[sd2196-bib-0010] Ferrera, I. (2017). Firms as political entities: Saving democracy through economic bicameralism. Cambridge, England: Cambridge University Press.

[sd2196-bib-0011] Frase, P. (2016). Four futures: Life after capitalism. London, England; New York, NY: Verso Books.

[sd2196-bib-0012] Galbraith, J. (2013). The third crisis in economics. Journal of Economic Issues, 47(2), 311–322.

[sd2196-bib-0013] Galbraith, J. (2014). The end of normal: The great crisis and the future of growth. New York, NY: Simon & Schuster.

[sd2196-bib-0014] Godley, W. , & Lavoie, M. (2007). Monetary economics – An integrated approach to credit, money, income, production and wealth. London, England: Palgrave Macmillan.

[sd2196-bib-0016] Gorz, A. (1999). Reclaiming work. Oxford, England: Blackwell/Polity Press.

[sd2196-bib-0017] Guardian . (2017). Finland trials basic income for unemployed. The Guardian. Retrieved from https://www.theguardian.com/world/2017/jan/03/finland-trials-basic-income-for-unemployed.

[sd2196-bib-0057] Guardian, (2018). Lessons from Europe's Biggest Basic Income Experiment. The Guardian, 10th August 2018, https://www.theguardian.com/world/2018/aug/10/lessons‐europe‐biggest‐basic‐income‐experiment‐upside‐newsletter.

[sd2196-bib-0018] Hartley, T. , van den Bergh, J. , & Kallis, G. (2020). Policies for equality under low or no growth: A model inspired by Piketty. Review of Political Economy, 32(2), 243–258. 10.1080/09538259.2020.1769293

[sd2196-bib-0019] Hickel, J. (2020). Less is more – How degrowth will save the world. London, England: Penguin.

[sd2196-bib-0020] Jackson, T. (2017). Prosperity without growth – Foundations for the economy of tomorrow. London, England: Routledge.

[sd2196-bib-0021] Jackson, T. (2019). The post‐growth challenge – Secular stagnation, inequality and the limits to growth. Ecological Economics, 156, 236–246. 10.1016/j.ecolecon.2015.03.019

[sd2196-bib-0022] Jackson, T. (2021). Post growth – Life after capitalism. Cambridge, England: Polity Press.

[sd2196-bib-0023] Jackson, T. , & Victor, P. (2016). Does slow growth lead to rising inequality? Ecological Economics, 121, 206–219. 10.1016/j.ecolecon.2018.10.010

[sd2196-bib-0024] JRF . (2015). Could a Citizen's income work? London: Joseph Rowntree Foundation. Retrieved from https://www.jrf.org.uk/sites/default/files/jrf/migrated/files/citizens-income-full.pdf.

[sd2196-bib-0055] Kaldor, N. (1955). Alternative theories of distribution. Review of Economic Studies, 23(2), 83–100.

[sd2196-bib-0025] Kalecki, M. (1939). Essays in the theory of economic fluctuations. Reprinted 2003. London, England: Routledge.

[sd2196-bib-0026] Kallis, G. , Paulson, S. , D'Alisa, G. , & Demaria, F. (2020). The case for degrowth. Cambridge, England: Polity Press.

[sd2196-bib-0027] Keynes, J. (1936). The general theory of employment, interest and money. London, England: Palgrave Macmillan.

[sd2196-bib-0028] Krusell, P , & Smith, A. (2014). Is Piketty's ‘second law of capitalism’ fundamental? Working paper (1st version). Washington DC: National Bureau of Economic Research.

[sd2196-bib-0029] Lazonick, W. (2017). The new Normal is “maximizing shareholder value”: Predatory value extraction, slowing productivity, and the vanishing American middle class. International Journal of Political Economy, 46(4), 216–226.

[sd2196-bib-0030] Mazzucato, M. (2018). The entrepreneurial state: Debunking public vs. private sector myths. London, England: Penguin.

[sd2196-bib-0031] McKay, A. (2001). Rethinking work and income maintenance policy: Promoting gender equality through a citizens' basic income. Feminist Economics, 7(1), 97–118. 10.1080/13545700010022721

[sd2196-bib-0032] Meade, J. (1988). Outline of economic policy for a labour government. In S. Howson (Ed.), The Collected Papers of James Meade vol.1, employment and inflation (p. 33). London, England: Unwin Hyman.

[sd2196-bib-0034] Minsky, H. (1986). Stabilizing an unstable economy. New Haven, CT: Yale University Press.

[sd2196-bib-0035] More, T. (1963). In P. Turner (Ed.), Utopia (1st Latin edition, Louvain, 1516), English translation (pp. 43–44). Harmondsworth, England: Penguin Classics.

[sd2196-bib-0036] Murray, C. (2008). Guaranteed income as a replacement for the welfare state. Oxford: Foundation for Law, Justice and Society. Retrieved from http://www.fljs.org/sites/www.fljs.org/files/publications/Murray.pdf.

[sd2196-bib-0037] ONS . (2014). Wealth and income 2010–2012. Statistical Bulletin. Office for National Statistics. Retrieved from http://www.ons.gov.uk/ons/dcp171778_368612.pdf.

[sd2196-bib-0038] ONS . (2017). Net capital stock to output ratio (TOTAL). London: Office for National Statistics. Retrieved from https://www.ons.gov.uk/economy/nationalaccounts/uksectoraccounts/timeseries/moo7/capstk.

[sd2196-bib-0039] Oxfam . (2015). Wealth: Having it all and wanting more. Oxford: OXFAM. Retrieved from http://policy-practice.oxfam.org.uk/publications/wealth-having-it-all-and-wanting-more-338125.

[sd2196-bib-0040] Piketty, T. (2010). On the long‐run evolution of inheritance: France 1820–2050. Paris: Paris School of Economics, Working Paper.

[sd2196-bib-0041] Piketty, T. (2014). Capital in the 21^st^ century. Cambridge, MA: Harvard University Press.

[sd2196-bib-0042] Piketty, T , Saez, E. , & Zucman, G. (2017). Distributional National Accounts: Methods and estimates for the United States data appendix. Retrieved from http://gabriel-zucman.eu/files/PSZ2018DataAppendix.pdf.

[sd2196-bib-0043] Pulkka, V. (2017). A free lunch with robots – Can a basic income stabilise the digital economy? Transfer, 23(3), 295–311.

[sd2196-bib-0044] Robinson, J. (1953). The production function and the theory of capital. Review of Economic Studies, 21, 81.

[sd2196-bib-0045] RSA . (2015). Creative citizen, creative state: The principled and pragmatic case for a universal basic income. London: Royal Society for the Arts. Retrieved from https://www.thersa.org/discover/publications-and-articles/reports/basic-income.

[sd2196-bib-0046] Stiglitz, J. (2015). The origins of inequality, and policies to contain it. National Tax Journal, 68(2), 425–448.

[sd2196-bib-0047] Storm, S. (2017). The New Normal: Demand, Secular Stagnation and the Vanishing Middle‐Class. INET Working Paper No 55, May 2017. Retrieved from https://www.ineteconomics.org/uploads/papers/WP_55-Storm-The-New-Normal.pdf.

[sd2196-bib-0054] Standing, G. (2017). Basic Income ‐ and how we can make it happen. London: Pelican.

[sd2196-bib-0048] Summers, L. (2014). U.S. economic prospects: Secular stagnation, hysteresis, and the zero lower bound. Business Economics, 49(2), 66–73.

[sd2196-bib-0049] Taylor, M. (2017). Good work: the Taylor review of modern working practices. London: Department for Business, Energy and Industrial Strategy (BEIS). Retrieved from https://www.gov.uk/government/publications/good‐work‐the‐taylor‐review‐of‐modern‐working‐practices.

[sd2196-bib-0053] Turner, A. (2015). Between Debt and the Devil: Money, Credit and Fixing Global Finance. Princeton: Princeton University Press.

[sd2196-bib-0050] Varoufakis, Y. (2016). The universal right to capital income. Project Syndicate. Retrieved from: https://www.project‐syndicate.org/commentary/basic‐income‐funded‐by‐capital‐income‐by‐yanis‐varoufakis‐2016‐10?barrier=accessreg.

[sd2196-bib-0051] Victor, P. (2019). Managing without growth: Slower by design not by disaster (2nd ed.). Cheltenham, England: Edward Elgar.

[sd2196-bib-0056] Wolf, M. (2015). The Shifts and the Shocks: What We’ve Learned – and Have Still to Learn – from the Financial Crisis. London: Penguin.

[sd2196-bib-0052] Wright, E. O. (2005). Basic Income as a Socialist Project. Paper presented at the annual US‐BIG Congress, University of Wisconsin 4–6 March 2005. Retrieved from http://www.ssc.wisc.edu/~wright/Basic%20Income%20as%20a%20Socialist%20Project.pdf.

